# Back to the future: evolutionary biology reveals a key regulatory switch in neuroblastoma pathogenesis

**DOI:** 10.1172/JCI167824

**Published:** 2023-05-15

**Authors:** Jaime N. Wertman, Jason N. Berman

**Affiliations:** 1 Department of Pediatrics, Izaak Walton Killam Health Centre and College of Pharmacy, Dalhousie University, Halifax, Nova Scotia, Canada.; 2 Children’s Hospital of Eastern Ontario Research Institute and Department of Pediatrics, University of Ottawa, Ottawa, Ontario, Canada.

## Abstract

While MYCN expression is an important contributing factor to heterogeneity in the natural history of neuroblastoma (NBL), a mechanistic understanding of this often mutationally quiet tumor has remained elusive. In this issue of the *JCI*, Weichert-Leahey and authors focused on the adrenergic and mesenchymal core regulatory circuitries (CRC) as NBL transcriptional programs. The authors previously showed that overexpression of LIM-domain-only 1 (LMO1), a transcriptional coregulator, synergizes with MYCN to accelerate tumor formation and metastasis in an NBL-zebrafish model. They now demonstrate experimentally, using genome-edited zebrafish, that a polymorphism in the human rs2168101 locus of the *LMO1* gene determines which CRC is active in a tumor. In some cases, LMO3 compensated for LMO1 loss and drove the adrenergic CRC in MYCN-positive NBL. This study exemplifies the value of evolutionary relationships and zebrafish models in the investigation of human disease and reveals pathways of NBL development that may affect prevention or intervention strategies.

## Epidemiology of neuroblastoma

Neuroblastoma (NBL) is the most common extracranial tumor in children. While it accounts for only 8%–10% of childhood cancers, NBL is responsible for 12%–15% of childhood cancer deaths. NBL arises most commonly in the adrenal gland from precursor neural crest cells, which ultimately give rise to adrenal chromaffin and sympathetic ganglion cells. However, NBL can arise anywhere along the sympathetic nervous system chain. The median age of diagnosis is 18 months. It is a remarkably heterogeneous disease with a clinical course that varies greatly depending on several risk-stratification factors (including age, stage, tumor histology, and amplification of the MYCN oncogene). Low-risk disease, typically seen in newborn infants or diagnosed prenatally, can exhibit spontaneous regression. In stark contrast, high-risk NBL is aggressive, with only a 40%–50% long-term overall survival. More than half of patients present with metastatic disease, occurring most commonly in the bone, bone marrow, and liver (1). Metastatic disease is the leading cause of death, and despite aggressive multi-model treatment, 5-year overall survival following recurrence of metastatic NBL is less than 8% (1, 2).

## Zebrafish models of neuroblastoma inform disease pathogenesis

Model organisms have been crucial tools in elucidating NBL biology and providing alternative avenues for therapeutic intervention. The vertebrate zebrafish benefits from the conservation of many human cancer genes and critical pathways regulating cell growth, proliferation, apoptosis, and differentiation (3, 4). Zebrafish also have conservation of key organs and tissues implicated in human cancers. Specific to NBL, zebrafish possess an interrenal gland, which is comparable to the human adrenal gland (5). Similarly, the bone marrow, a frequent site of NBL metastasis, exists in the zebrafish as the kidney marrow (distinct from the interrenal gland) (6). Zebrafish also have advantages over rodents with respect to large numbers of externally fertilized offspring and rapid embryonic development (4). These features allow for the manipulation of gene networks and developmental processes not easily performed in mammals, in an animal amenable to direct visualization by microscopy.

The first zebrafish model of NBL was published in 2012 and employed a transgenesis strategy whereby human MYCN was driven by dopamine-β-hydroxylase gene promoter (5). These fish developed neuroendocrine tumors resembling human NBL in the interrenal gland, the onset of which could be accelerated by coexpression of anaplastic lymphoma kinase (ALK), which is found to be mutationally activated in approximately 15% of high-risk NBL cases (7, 8). This model was subsequently leveraged by these same authors to determine a role for *GAB2* (encoding GRB2-associated binding protein-2) in NBL pathogenesis, through the activation of SH2-containing protein tyrosine phosphatase-2 (SHP2) and the RAS pathway. Later, this model was used to lay the foundation for the current study, providing definitive evidence that LIM-domain-only 1 (LMO1) synergizes with MYCN to result in a more penetrant and aggressively metastatic disease (9).

## LMO1 is a critical factor determining neuroblastoma aggressiveness

**Figure 1:**
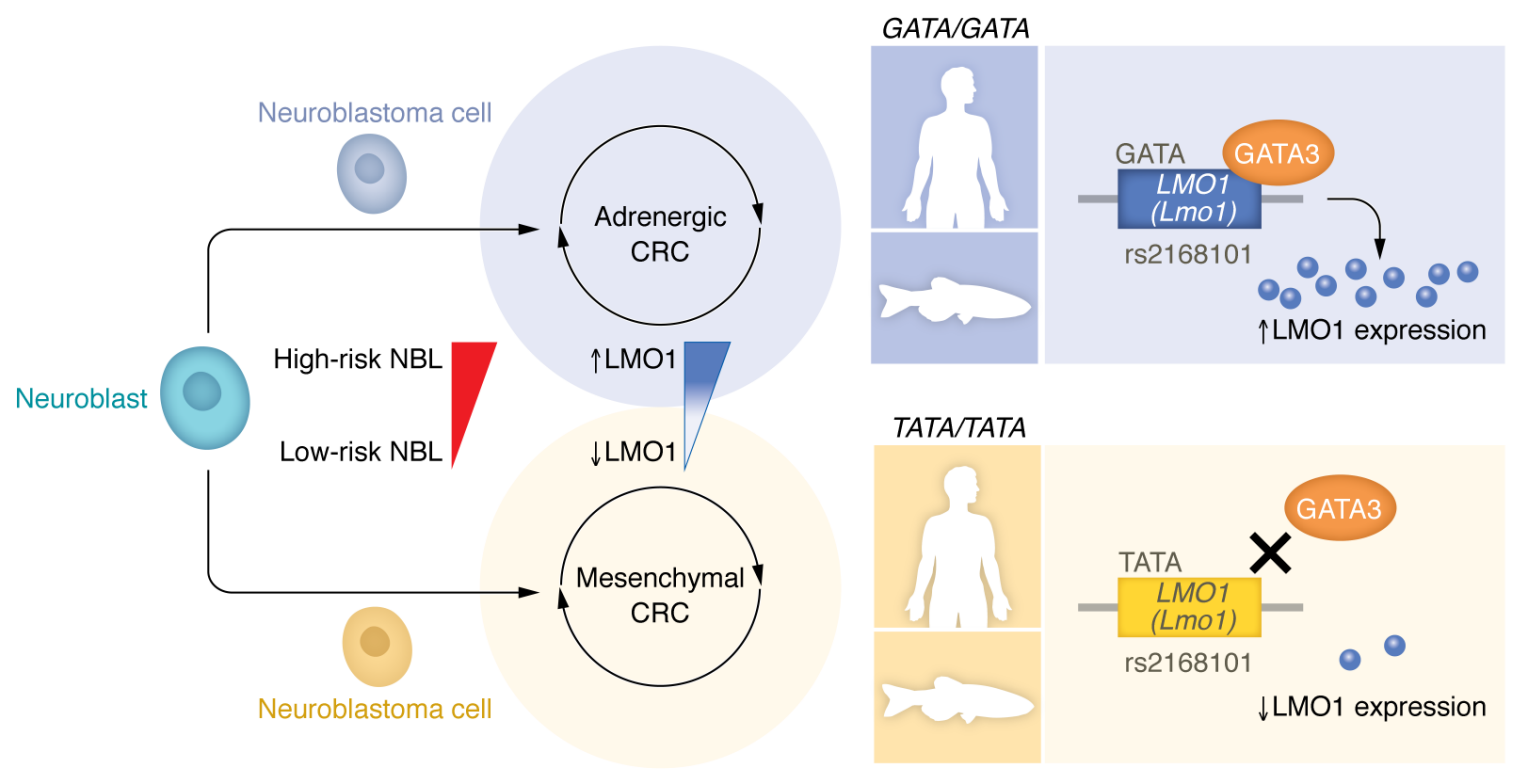
The rs2168101 G → T transversion in *LMO1* reduces GATA3 binding, resulting in lower LMO1 levels and reliance on the mesenchymal CRC. NBL cells arise from neural crest cells during development. The adrenergic and mesenchymal CRCs are the main autoregulatory transcriptional loops involved in NBL. LMO1 is a coregulator in the adrenergic CRC. The presence of a G allele at the rs2168101 SNP results in a GATA-binding motif in this intron, promoting GATA3 binding and creating a super enhancer that drives high levels of LMO1 expression. When replaced by a T, this TATA sequence prevents GATA3 binding, resulting in lower LMO1 levels and lower NBL risk. Transgenic zebrafish lines harboring this SNP replicate findings in humans.

In this issue of the *JCI*, Weichert-Leahey et al., (10) have returned to their original zebrafish NBL model to validate findings from a genome-wide association study (GWAS) analysis and reveal how core regulatory circuitries (CRC) underpin the initial development of NBL (Figure 1). The authors previously identified a G → T polymorphism at the rs2168101 locus within the first intron of the *LMO1* gene, which predisposes the carrier to NBL when the guanine base is present (11). By contrast, the T allele was found to be protective, but is only present in humans. Exploiting this evolutionary finding, the authors recreated this polymorphism in the zebrafish *lmo1* ortholog using TALEN-mediated gene editing. By subsequently crossing this mutant line with their established transgenic NBL-susceptible zebrafish line, *Tg(dβh:MYCN;dβh:EGFP)*, they were able to experimentally demonstrate the protective effect of the T allele in the formation of NBL. Similar levels of protection were obtained when the *MYCN*-expressing line was crossed with a novel *lmo1* knockout zebrafish, highlighting the role of lmo1 as critical for neuroblastomagenesis. Despite not being a transcription factor itself, lmo1 was identified as the key regulatory switch that, as a consequence of GATA3 binding, creates a super enhancer that governs which CRC is activated: the more lineage-committed pro-NBL adrenergic CRC program or the less differentiated mesenchymal CRC program. Importantly, these CRC gene signatures are consistent across human NBL tumors (mesenchymal in low risk and adrenergic in high risk) and LMO3, an LMO1 paralog, can sometimes substitute for LMO1 and drive the more aggressive adrenergic CRC program.

## Translational relevance and opportunities

This story is a remarkable one that is emblematic of the opportunities inherent in the zebrafish model to inform human cancer biology. While the identification of these CRCs in NBL was previously ascertained (12–14), their link back to LMO1 as the upstream driver was only realized through zebrafish modeling. This discovery can further refine prognostic scoring in NBL, which could help patients with low-risk disease avoid the potentially life-threatening adverse events that can occur with chemotherapy. The identification of additional NBL-promoting alleles can help pave the way for genetic screening and surveillance protocols for early detection of NBL, much like what has been successfully undertaken for other cancer predisposition disorders like Li-Fraumeni syndrome (15). Converting the G allele to a protective T allele to either prevent NBL or convert high-risk to low-risk disease is also a tantalizing prospect in the current era of emerging gene therapies.

A limitation of this study, acknowledged by the authors, is the focus specifically on the NBL cell population for transcriptional analysis, which does not take into consideration the potential contribution of the surrounding niche. The tumor microenvironment in NBL includes immune cells, extracellular matrix, and soluble factors (16). Primary NBL gene–expression data shows an enrichment of receptors, such as the integrin α5β1 and CD44 that are important cell structural components (17). Moreover, other receptors important for cell homing, including CXCR4 and CXCR7, may be expressed at various levels, affecting metastatic spread (18). In the future, patient-derived xenografts in humanized mice — and in recently described humanized zebrafish (19) — that incorporate both NBL cells and components of the surrounding stroma, will provide even more comprehensive preclinical platforms in which to evaluate therapeutic interventions in the context of CRC programs and cell migration patterns.

## Conclusion

In this study (10), Weichert-Leahey and authors expand upon their discovery of an NBL-susceptible polymorphism in the human population. They experimentally model the G → T polymorphism in the zebrafish, resulting in GATA or TATA allele patterns, and demonstrate a consistent genotype-phenotype correlation regarding NBL onset. The zebrafish model is subsequently exploited as a tool for dissecting a critical regulatory function for LMO1 that is conserved between fish and human NBL tumors. This work sheds light on the molecular underpinnings of NBL development and opportunities for enhanced risk stratification, disease prediction, and, perhaps, even earlier detection and prevention or intervention. Through their unexpected use of evolutionary biology in the context of the fast-paced technology-driven study of human health and disease, the authors have left the reader with a broader compelling message: advances in science often rely on lessons learned from the past.

## Version 1 05/15/2023

Electronic publication
